# Haemoadsorption reduces the inflammatory response and improves blood flow during ex vivo renal perfusion in an experimental model

**DOI:** 10.1186/s12967-017-1314-5

**Published:** 2017-10-25

**Authors:** Sarah A. Hosgood, Tom Moore, Theresa Kleverlaan, Tom Adams, Michael L. Nicholson

**Affiliations:** Department of Surgery, University of Cambridge, Addenbrooke’s Hospital, Cambridge, Hill’s Road, CB2 OQQ UK

**Keywords:** Kidney, Ex vivo perfusion, Haemoadsorption, Inflammation

## Abstract

**Background:**

Ex-vivo normothermic perfusion strategies are a promising new instrument in organ transplantation. The perfusion conditions are designed to be protective however the artificial environment can induce a local inflammatory response. The aim of this study was to determine the effect of incorporating a Cytosorb adsorber into an isolated kidney perfusion system.

**Methods:**

Porcine kidneys were subjected to 22 h of cold ischaemia then reperfused for 6 h on an ex vivo reperfusion circuit. Pairs of kidneys were randomised to either control (n = 5) or reperfusion with a Cytosorb adsorber (n = 5) integrated into the circuit. Tissue, blood and urine samples were taken for the measurement of inflammation and renal function.

**Results:**

Baseline levels of cytokines (IL-6, TNFα, IL-8, IL-10, IL-1β, IL-1α) were similar between groups. Levels of IL-6 and IL-8 in the perfusate significantly increased during reperfusion in the control group but not in the Cytosorb group (P = 0.023, 0.049). Levels of the other cytokines were numerically lower in the Cytosorb group; however, this did not reach statistical significance. The mean renal blood flow (RBF) was significantly higher in the Cytosorb group (162 ± 53 vs. 120 ± 35 mL/min/100 g; P = 0.022). Perfusate levels of prostaglandin E2 were significantly lower in the Cytosorb group (642 ± 762 vs. 3258 ± 980 pg/mL; P = 0.0001). Levels of prostacyclin were significantly lower in the Cytosorb group at 1, 3 and 6 h of reperfusion (P = 0.008, 0.003, 0.0002). Levels of thromboxane were also significantly lower in the Cytosorb group throughout reperfusion (P = 0.005). Haemoadsorption had no effect on creatinine clearance (P = 0.109).

**Conclusion:**

Haemoadsorption can reduce the inflammatory response and improve renal blood flow during perfusion. Nonetheless, in this model haemoadsorption had no influence on renal function and this may relate to the broad-spectrum action of the Cytosorb adsorber that also removes potentially important anti-inflammatory mediators.

## Background

The adoption of ex vivo normothermic perfusion technologies in clinical transplantation offers significant advantages compared to hypothermic techniques [1–3]. Restoring function ex vivo upregulates protective mechanisms [4], replenishes cellular energy [5] and allows a functional assessment of an organ prior to transplantation [6]. Although organs are perfused in a protective environment without leukocytes or complement, inflammatory processes are upregulated [4]. This is possibly due to the mechanical process of perfusion and direct contact of the perfusate with artificial surfaces. The consequences of this heightened inflammatory response in this setting are unknown. Although they appear not to have a detrimental effect on graft outcome, removing them during perfusion may be beneficial. Inflammatory mediators play an important role in exacerbating the severity of renal ischaemia reperfusion injury (IRI) [7]. IRI is a complicated multifactorial process causing damage to the vascular endothelial cells and tubular epithelium due to the production of reactive oxygen species (ROS), upregulation of pro-inflammatory cytokines, recruitment of neutrophils, and complement and platelet activation [8–10]. In transplantation, periods of warm and cold ischaemia exacerbate IRI causing graft dysfunction and reducing graft survival [11, 12].

The removal of cytokines using haemoadsorption has been advocated in the management of severe inflammatory-driven disease states such as sepsis [13] and severe systemic inflammatory response syndrome (SIRS) [14–16]. Although haemoadsorption effectively reduces the concentration of circulating cytokines and improves survival, its effectiveness in other circumstances, such as during cardiopulmonary bypass, remains inconclusive [17].

Locally within the kidney, circulating cells and tubular epithelial cells produce numerous cytokines. Pro-inflammatory cytokines such as IL-1β, IL-6, TNFα and IL-8 are expressed in the early revascularisation phase after transplantation [18–20]. They orchestrate a network of interlinked responses causing tissue damage. The Cytosorb adsorber is most effective for removing molecules in the 10–50 kDa range, which includes many anti- but also pro-inflammatory cytokines [21]. Therefore, the overall effect of the Cytosorb adsorber will depend on the balance between removal of beneficial and deleterious mediators. The aim of this study was to examine the effect of cytokine haemoadsorption in an isolated kidney perfusion system.

## Methods

### Kidney retrieval

Under Home Office ‘The Animals’ (Scientific Procedures) Act 1986 in the UK, five landrace cross pigs weighing 61.6 ± 7.9 kg underwent a general anaesthesia and midline laparotomy. Both renal pedicles were exposed and the kidneys dissected. A bolus injection of 25000 IU heparin was given 5 min before the renal artery and renal veins were ligated and the kidneys removed. One litre of blood was collected from the aorta into two citrate–phosphate-dextrose-adenine blood bags before the animal was culled with an overdose of barbiturate.

### Preservation

Immediately after retrieval the kidneys were flushed with 500 mL of ice-cold University of Wisconsin (UW) preservation solution at a hydrostatic pressure of 100 mmHg. Kidneys were then placed in bags containing ice-cold UW solution, packed in ice and stored for 22 h.

### Ex-vivo perfusion system

After removal from cold storage the kidneys were weighed and prepared for reperfusion on the ex vivo perfusion circuit. The renal artery, vein and ureter were cannulated and the kidney was flushed with 200 mL of cold Ringer’s solution to remove the preservation solution.

Perfusion was carried out using an adapted paediatric cardiac bypass system (Medtronic, Bioconsole 560) as described previously [4, 5]. The ex vivo perfusion system was primed with 300 mL Ringer’s solution (Baxter Healthcare, Thetford UK), 2.5 g Mannitol (Baxter Healthcare), 12 mL sodium bicarbonate 8.4% (Fresenius Kabi, Runcorn, UK) and 3000 IU heparin (LEO Pharma A/S, Ballerup, Denmark). Whole blood (300 mL) was then added and recirculated to a temperature of 37.4 °C. The blood-based solution was oxygenated with a balance of 95% oxygen/5% CO_2_ at a flow rate of 0.1 L/min.

The blood-based solution was circulated continually through the kidney via the renal artery at a mean arterial pressure of 85 mmHg and pump speed of 1500 RPM. A nutrient solution (Synthamin 17 10%, Baxter Healthcare) with 15 mL of sodium bicarbonate 8.4% and 100 IU of insulin added (Actrapid, Novo Nordisk, London, UK) was infused at a rate of 20 mL/h. Glucose 5% (Baxter Healthcare) was infused at a rate of 5 mL/h and Ringer’s solution was used to replace urine output (mL for mL).

### Study design

A computerised sequence was generated and pairs of kidneys randomised to either control or Cytosorb adsorber (Cytosorb, LINC Medical Systems Ltd, Leicester, UK) (n = 5 kidneys per group).

**Fig. 1:**
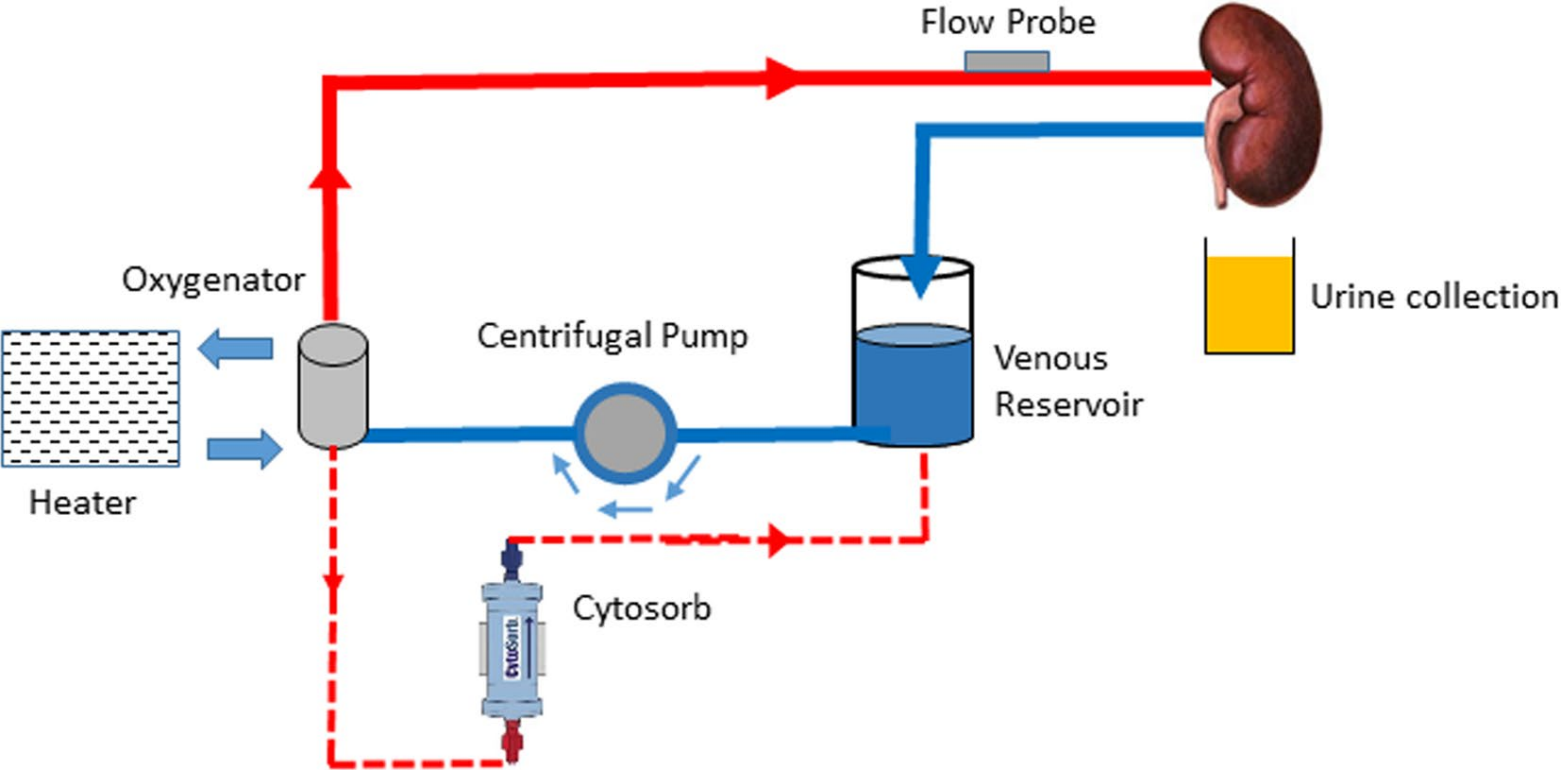
A schematic diagram of the perfusion circuit. The blood based perfusate circulates from the venous reservoir into the centrifugal pump. It is pumped into the membrane oxygenator at a set pump speed and mean arterial pressure were it is oxygenated and warmed to 37.4 °C before entering into the arterial arm of the circuit and into the kidney via the renal artery. The Cytosorb was attached to a line from the oxygenator then fed back into the venous reservoir allowing a collateral circulation of the perfusate without altering the renal blood flow

The Cytosorb adsorber was flushed and primed with Ringer’s solution then added to the circuit by connecting it to the line from the oxygenator allowing the blood to flow through the adsorber back into the venous reservoir in parallel with the main flow to the renal artery (Fig. 1).

### Outcome measures

The renal blood flow (RBF) was recorded every 5 min for the 30 min and thereafter every 30 min. Samples of perfusate (arterial and venous) were collected pre-perfusion then hourly. Urine samples were collected hourly.

Samples of perfusate and urine were sent to biochemistry for the measurement of urea and electrolytes. Samples of perfusate were also sent for haematology analysis.

Samples of venous perfusate and urine were centrifuged at 1600 rpm for 10 min at 4 °C. The supernatant was collected and frozen in liquid nitrogen then stored at − 70 °C until analysed.

Samples of arterial and venous perfusate were collected at 1, 3 and 6 h of reperfusion for blood gas analysis (OPTI-CCS). Oxygen consumption was calculated (arterial PaO_2_ – venous PaO_2_ × RBF/weight of kidney).

### RNA extraction and relative gene expression

Core biopsies were taken from each kidney (n = 10) in situ (baseline) and after 6 h of perfusion. Biopsies were stored in RNA later solution (Invitrogen RNA later™ Soln.) for fixing. The tissue was lysed (Precellys Lysing Kit MK28-R) and RNA was extracted (Invitrogen PureLink™ RNA Mini Kit & Invitrogen TURBO DNA-free™ Kit). The RNA was used to generate the cDNAs for each sample by reverse transcription (Applied Biosystems High Capacity cDNA Reverse Transcription Kit). Primers for ICAM-1, IL-1β, IL-6, IL-8, HMGB-1, TLR4 and RPL4 were designed using NCBI Primer Blast (Appendix: Table 2). Relative qPCR (Stratagene Mx3005P) was then performed using SYBR Green (Applied Biosystems *Power* SYBR^®^ Green PCR Master Mix) on baseline and 6 h perfusion samples for all kidneys and all genes. Cycling conditions are shown in Appendix: Table 3. Samples were assayed in triplicate and Ct values were averaged for each gene. The housekeeping gene (RPL4) Ct value was subtracted from the average Ct value of other genes within that sample (ΔCt). Since the kidneys were paired, the control ΔCt value was subtracted from the Cytosorb adsorber ΔCt value (ΔΔCt). The relative fold change for each gene between treatment and control was then calculated using 2^−ΔΔCt^. The same analysis was performed comparing the relative expression difference within each kidney between baseline and 6 h.

### Protein analysis

Perfusate levels of the following cytokines and inflammatory/injury markers were measured by ELISA as per the manufacturer’s instructions; IL-1α, IL-1β, IL-6, IL-8, IL-10, TNFα (R&D Systems), CRP (R&D Systems Porcine C-Reactive Protein/CRP) and neutrophil gelatinase-associated lipocalin (NGAL) (Elabscience Porcine NGAL ELISA). Perfusate levels of IL-1RA (Elabscience Porcine IL-1RA ELISA Kit) Prostaglandin E_2_ Assay, prostacyclin (PG12) and thromboxane B1 (R&D Systems), HMGB1 (Cusabio Pig High Mobility Group Protein B1), heme (BioVision Heme Colorimetric Assay Kit) and urinary levels of NGAL were also measured.

### Statistics

Values are presented as mean ± standard deviation. Continuous variables such as RBF were plotted against time. Values were compared using a paired t test. P ≤ 0.050 was considered statistically significant. Statistical analysis was performed using Microsoft Excel and Graphpad Prism 7 (GraphPad Software Inc., La Jolla, CA, USA).

## Results

The mean cold ischaemic times in the Cytosorb and control groups were 21.7 ± 0.3 h and 22.9 ± 0.3 h respectively (P = 0.196).

### Haemodynamics

The haematocrit (HCT) and platelet count were significantly lower in the Cytosorb group at the start of perfusion compared to the control (P = 0.046, 0.008, 0.003, respectively; Table 1). At 6 h there was no significant difference in the HCT or Hb counts (P = 0.650, 0.626, 0.444, respectively; Table 1). However, the platelet count was significantly lower in the Cytosorb group (P = 0.0003), falling by 50% compared with 40% in the control during perfusion.

**Table 1:** Haemoglobin (Hb), haematocrit (HCT), platelet count, white cell count (WCC) and heme before and after 6 h of reperfusion

Parameters	Pre-perfusion			After 6 h of perfusion		
	Control	Cytosorb	P value	Control	Cytosorb	P value
Hb (g/L)	45.2 ± 6.5	38.0 ± 5.2	0.115	37.0 ± 9.5	34.6 ± 7.6	0.626
HCT (L/L)	0.16 ± 0.02	0.13 ± 0.01	0.046*	0.14 ± 0.03	0.13 ± 0.02	0.845
Platelets (10 × 9/L)	172 ± 27	106 ± 22	0.008*	106 ± 28	52 ± 26	0.003*
WCC (10 × 9/L)	2.6 ± 0.9	2.4 ± 0.1	0.611	1.7 ± 1.4	1.2 ± 0.7	0.262
Heme (pmol/L)	33.2 ± 1.6	33.1 ± 1.3	0.785	38.8 ± 9.3	67.6 ± 56.1	0.342

Kidneys were reperfused with autologous blood for 6 h on an ex vivo circuit * P <0.050

Baseline levels of heme in the perfusate were similar between groups (P = 0.785; Table 1). There was a numerical increase in the control group during perfusion (P = 0.356) and levels were numerically higher in the control group compared to the Cytosorb group at 6 h (P = 0.342; Table 1).

**Fig. 2:**
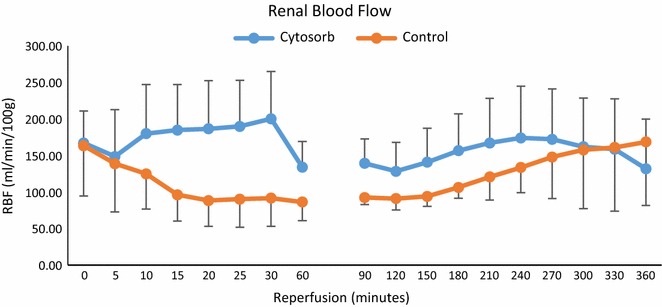
Mean renal blood flow during 6 h of reperfusion in the control and Cytosorb groups

At the start of perfusion the mean RBF was similar between the groups (Fig. 2). In the control group during the first 20 min of perfusion, the RBF fell and then remained stable before starting to increase after 3 h. In the Cytosorb group there was an increase in the RBF over the first 30 min of perfusion. The RBF then fell abruptly remaining stable before gradually recovering at 2 h (Fig. 2). The mean RBF was significantly lower in the control group (control 120 ± 35 vs. Cytosorb 162 ± 52 mL/min/100 g; P = 0.022).

Levels of oxygen consumption were significantly higher in the Cytosorb group at 1 h (96.4 ± 28.1 vs. 59.1 ± 17.2 mL/min/g; P = 0.027) and numerically higher at 3 h (104.4 ± 31.1 vs. 72.0 ± 8.7 mL/min/g; P = 0.057) of perfusion. There was no significant difference in levels at 6 h (P = 0.132).

### Renal function

There was no significant difference in the total amount of urine produced between the groups (control 1321 ± 497 mL vs. Cytosorb 1462 ± 515 mL; P = 0.644). Levels of creatinine clearance were similar between groups [area under the curve (AUC) control 31.7 ± 12.3 vs. Cytosorb 40.5 ± 17.9 mL/min/100 g; P = 0.109]. Tubular function was also similar between groups (AUC fractional excretion of sodium, control 219 ± 60.3 vs. Cytosorb 202 ± 93%; P = 0.561).

### NGAL

Levels of NGAL in the perfusate were numerically lower in the Cytosorb group at 6 h but did not reach statistical significance (Cytosorb 18 ± 7 vs. control 107 ± 77 ng/mL; P = 0.055). However, urinary NGAL levels were significantly lower in the Cytosorb group throughout reperfusion (6 h 1.1 ± 1.7 vs. 173.5 ± 115.5 ng/mL; P = 0.030).

### Cytokines

**Fig. 3:**
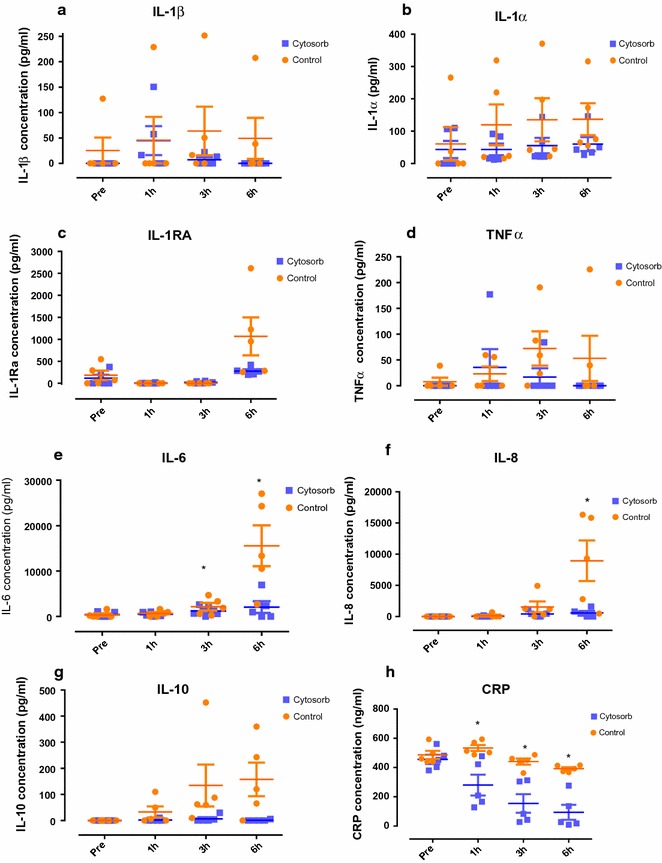
Perfusate levels of **a** IL-1β, **b** IL-1α, **c** IL-RA, **d** TNFα, **e** IL-6, **f** IL-8, **g** IL-10, and **h** CRP pre, 1, 3 and 6 h of reperfusion in the control and Cytosorb groups measured by ELISA. *P < 0.05

Baseline levels of cytokines (IL-1β, IL-1α, IL-receptor antagonist (IL-RA), TNFα, IL-6, IL-8, IL-10 and C-reactive protein (CRP) were similar among groups (P > 0.05). There was a numerical increase in the levels of IL-1β, IL-1α, IL-RA, TNFα and IL-10 in the control group throughout perfusion, whereas levels remained low in the Cytosorb group. Levels of IL-6 were significantly lower in the Cytosorb group at 3 and 6 h of perfusion (P = 0.040, 0.023, respectively, Fig. 3). Levels of IL-8 were numerically lower at 6 h of perfusion in the Cytosorb group (P = 0.052). CRP was significantly lower in the Cytosorb group at 1, 3 and 6 h perfusion. There was no significant difference in levels of IL-1β, IL-1α, IL-RA and IL-10 between the groups at 1, 3 and 6 h of perfusion (P > 0.05). The Cytosorb adsorber reduced IL-1β by 39 ± 54%, IL-1α by 48 ± 25%, IL-RA by 52 ± 67%, TNFα by 39 ± 54%, IL-6 by 87 ± 12%, IL-8 by 59 ± 54%, IL-10 by 40% and CRP by 75 ± 31% after 6 h of perfusion compared to the control group.

### Prostaglandins

**Fig. 4:**
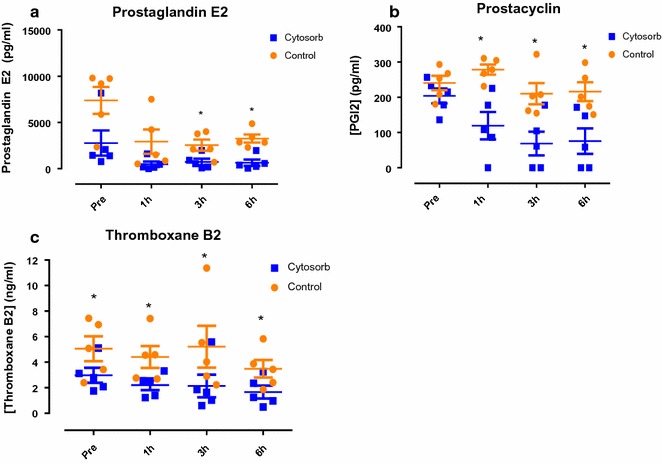
Perfusate levels of **a** Prostaglandin E2 (PGE2), **b** Prostacyclin I2 (PGI2), and **c** Thromboxane B2 (TXB2) measured by ELISA. *P < 0.05

Baseline levels of prostaglandin E2 and prostacyclin were similar in both groups (P = 0.166, 0.236, respectively; Fig. 4), whereas baseline levels of thromboxane were significantly lower in the Cytosorb group (P = 0.024; Fig. 4). There was a numerical decrease in levels of prostaglandin E2 in both groups during perfusion (control P = 0.11, Cytosorb P = 0.066; Fig. 4a) and levels were significantly lower in the Cytosorb group compared to controls at 3 and 6 h (P = 0.023, 0.001; Fig. 4a). Levels of prostacyclin fell significantly in the Cytosorb group (P = 0.033; Fig. 4b) but there was only a numerical fall in the control group (P = 0.095; Fig. 4b). Prostacyclin levels were significantly lower in the Cytosorb group at 1, 3 and 6 h of perfusion compared to controls (P = 0.008, 0.003, 0.0002; Fig. 4b). Levels of thromboxane B2 were significantly lower in the Cytosorb group throughout perfusion (1 h P = 0.035, 3 h P = 0.025 and 6 h P = 0.005; Fig. 4c).

### Gene expression

**Fig. 5:**
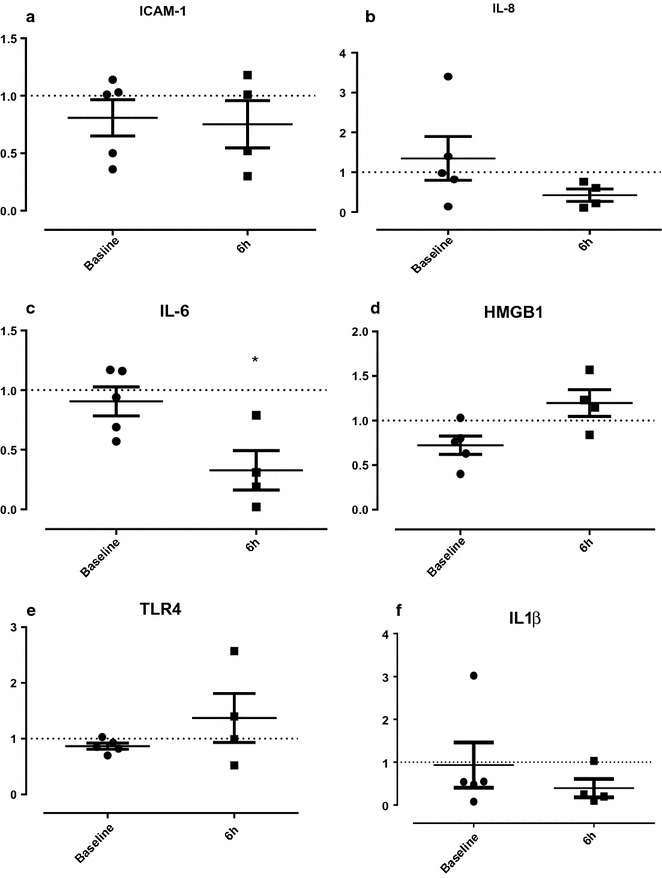
Gene expression in the Cytosorb group relative to the control group after 6 h reperfusion, normalised to RPL4. The relative fold change for each gene between treatment and control was calculated using 2^−ΔΔCt^. The same analysis was performed comparing the relative expression difference within each kidney between in situ (baseline) and 6 h. *P < 0.05

Paired kidney analysis showed a significant downregulation of renal tissue levels of IL-6 (P = 0.017; Fig. 5) in the Cytosorb group relative to the control group after 6 h of perfusion. There was also a numerical decrease in renal IL-8 expression at 6 h in the Cytosorb group relative to the control.

Individual kidney analysis of gene expression at 6 h relative to baseline showed a statistically significant increase in IL-6 (P = 0.017), but there were no differences in IL-8 (P = 0.176) and IL-1β (P = 0.448) in the control group relative to the adsorber.

## Discussion

The Cytosorb adsorber has been used widely in the clinic with the main indication for the treatment of sepsis [21]. Numerous studies have shown that it is a safe and effective way of reducing the systemic inflammatory response. Suppression of inflammation by inhibiting specific inflammatory mediators can reduce the effects of IRI [22]. Haemoadsorption has the advantage that a range of the pro-inflammatory cytokines are effectively reduced rather than targeting individual cytokines. This had a number of beneficial effects in this isolated kidney model. During perfusion there was an increase in the level of circulating pro-inflammatory cytokines, particularly IL-6 and IL-8 after 3 h, but haemoadsorption significantly abrogated this effect. IL-6 and IL-8 are produced as a result of NFκB signalling, typically initiated via IL-1β and TNFα dependent pathways [23]. There was also a significant reduction in IL-6 gene expression and a numerical decrease in IL-8, suggesting a sustained effect in altering the pro-inflammatory profile. However, longer follow up would be needed to determine the full effect of this. Levels of IL-1β and TNFα also increased during reperfusion, although to a lesser extent. This may reflect the study of an isolated perfused organ as opposed to a whole organism, where a larger systemic effect might be anticipated. Nonetheless, haemoadsorption resulted in a numerical reduction in circulating levels.

The Cytosorb adsorber is not specific for pro-inflammatory cytokines and as a consequence the anti-inflammatory cytokine IL-10 along with the receptor antagonist IL-1RA were also reduced by haemoadsorption. IL-10 is essential for maintaining the integrity and homeostasis of tissue epithelial layers and suppressing the pro-inflammatory response [24, 25]. Receptor antagonists inhibit the binding of their corresponding cytokines to prevent their action [26]. IL-1RA can reduce the expression of intracellular adhesion molecule-1 (ICAM-1), and reduce apoptosis [24, 25]. This may explain why we found no difference in the expression of ICAM-1, other pro-inflammatory cytokines or TLR-4 after 6 h of perfusion. Further investigation into the balance of pro- and anti-inflammatory mediators is necessary.

The results of haemoadsorption in the treatment of other inflammatory states such as that induced by cardiopulmonary bypass has been variable. A randomised controlled trial demonstrated no significant differences in pro-inflammatory cytokine levels and no effect on patient outcome [16]. One explanation for the lack of effect may be the duration of the bypass procedure. Patients underwent bypass for a median of 191 min with cytokine levels peaking at 2 h after treatment. Our data show that the majority of cytokines are upregulated after 3 h of perfusion. Therefore, the addition of the Cytosorb adsorber during cardiopulmonary bypass may be beneficial for more prolonged cases.

In spite of the reduction of pro-inflammatory cytokines in this study, haemoadsorption had no effect on renal function. All kidneys demonstrated a similar level of glomerular and tubular function. NGAL, a reliable marker of proximal tubular damage [26, 27], was high in the control kidneys corresponding to the level of cold ischaemic injury. NGAL was reduced in the Cytosorb group, but rather than indicating a reduction in injury during reperfusion this was probably due to direct haemoadsorption of the NGAL molecule, which has a molecular mass of 23 kDa.

The vascular endothelium plays an important role in regulating RBF. In response to ischaemic injury there is a reduction in vasodilatory mediators such as prostacyclin and prostaglandin E2 and enhanced expression of thromboxane, which promotes vasoconstriction, thus reducing blood flow [28]. A reduction in the RBF and oxygen consumption was evident in the control group throughout perfusion and this corresponded with higher thromboxane levels. Haemoadsorption improved overall blood flow throughout 6 h of reperfusion, but at 30 min there was a noticeable rapid decrease in blood flow. This may be explained by the finding of lower levels of thromboxane and a fall in prostacyclin and prostaglandin E2 in the first hour of haemoadsorption. This suggests that the beneficial effect of filtering thromboxane was counteracted by the filtration of prostacyclin and prostaglandin E2.

Platelet activation can enhance IRI and reduce blood flow through endothelial adhesion and release of vasoconstriction mediators [29]. The platelet count was significantly lower at baseline in the Cytosorb group due to the extra volume required to fill the adsorber. This may have enhanced the blood flow at the beginning of perfusion. Nonetheless, the platelet count fell in both the Cytosorb and control groups but the decrement was 10% greater after haemoadsorption and this may be another factor in improving RBF. Heme is associated with the activation of pro-inflammatory, apoptotic and oxidant pathways and can result in further organ damage [30] but in this study was not significantly reduced by haemoadsorption.

Cytokine filtration has recently been applied to ex vivo lung perfusion [31]. Iskender et al. demonstrated that porcine lungs perfused ex vivo for 12 h with the Cytosorb adsorber incorporated into the circuit developed less oedema, had improved electrolyte balance and lower neutrophil infiltration [31]. This present study suggests that haemoadsorption could be beneficial to the kidney during ex vivo perfusion. Ex-vivo normothermic perfusion has recently been introduced into clinical practice in kidney transplantation [1]. Adding a Cytosorb adsorber to the system would enable a proportion of the inflammatory mediators to be removed. However, supplementation particularly of vasodilatory mediators would be necessary to maintain a stable environment. We chose to examine the effect of adding the adsorber into the circuit in the most practical and convenient way. Whole blood rather than leucocyte depleted or packed red cells was used to determine the full effect of the adsorber. This provided important information on the preservation conditions, particularly in regard to the removal of prostaglandins. A single centrifugal pump circulated the blood based solution at a set pump speed and pressure. The Cytosorb was placed in the arterial side of the circuit from the membrane oxygenator back into the reservoir forming a collateral circulation. The adsorber may be more effective if it was placed on the venous arm of the system. However, this would require the addition of another pump which may alter the perfusion of the kidney. The degree of reduction in the levels of cytokines was within range of the described effects of the Cytosorb and with the low volume in the system it is likely that the blood based solution did effectively pass through the adsorber.

## Conclusion

In an isolated renal perfusion model, haemoadsorption reduced the inflammatory response and improved RBF. Nonetheless, it had no influence on renal function and this may relate to the broad-spectrum action of the Cytosorb adsorber that also removes potentially important anti-inflammatory mediators.

## Appendix

See Tables 2, 3.

**Table 2:** qPCR primers

Gene	NCBI accession number	Primer sequences
ICAM-1	NM_213816.1	TCAATGTGGCCCCTAAACACC
		GTCTCTAGGCCAAAGCTGGT
IL-1β	NM_214055.1	CCTTGAAACGTGCAATGATGACT
		GCCAGCCAGCACTAGAGATT
IL-6	NM_001252429.1	GGGTTCAATCAGGAGACCTGC
		CGGCCTCGACATTTCCCTTA
IL-8	NM_213867.1	AGAGTGGACCCCACTGTGAA
		TGTTGTTGCTTCTCAGTTCTCTT
HMGB-1	NM_0010040134.1	GCTCAGAAAGGTGGAAGACCAT
		TCATAACGGGCCTTGTCCG
RPL4	XM_005659862.2	TGAGCTCTATGGCACTTGGC
		GAATCTTCTTGCGTGGTGCG
TLR4	NM_001113039.2	TCATGCTTTCTCCGGGTCAC
		TAGGAACCACCTGCACGCAA

**Table 3:** PCR thermocycler conditions

Segment	Temperature (°C)	Time (s)
Segment 1 1 cycle	95	600
Segment 2 40 cycles	95	30
	59	60
Segment 3 1 cycle	95	60
	59	30
	95	30
